# Beyond Peptic Ulcers: Oesophageal Haematoma, an Under-Recognised Cause of Gastrointestinal (GI) Bleeding on Dual Antiplatelet Therapy (DAPT)

**DOI:** 10.7759/cureus.88563

**Published:** 2025-07-23

**Authors:** Umesh Kumar Pabani, Seena Darwin Nirmala, Subirna Visvalingam, Moska Rasoul, Shah R Mohdnazri

**Affiliations:** 1 Internal Medicine, Southend University Hospital, Southend-on-Sea, GBR; 2 Cardiology, Southend University Hospital, Southend-on-Sea, GBR; 3 Acute Medicine, Southend University Hospital, Southend-on-Sea, GBR; 4 Cardiology, Mid and South Essex NHS Foundation Trust, Southend University Hospital, Southend-on-Sea, GBR

**Keywords:** chest pain, clopidogrel, dual antiplatelet therapy, gastrointestinal bleeding, oesophageal haematoma, percutaneous coronary intervention

## Abstract

Dual antiplatelet therapy (DAPT) is widely used for secondary prevention following acute coronary syndrome and percutaneous coronary intervention (PCI). Gastrointestinal (GI) bleeding is a known complication of DAPT, typically due to peptic ulcer disease or gastritis. However, oesophageal haematoma is a rare and under-recognised cause of upper gastrointestinal bleeding (UGIB) in this setting. We present a case of a 65-year-old woman who attended an Accident and Emergency Department with typical central chest pain, vomiting, and haematemesis, six months after undergoing PCI and commencing DAPT. Initial investigations raised suspicion for acute coronary syndrome as she presented with chest pain, but coexistent persistent gastrointestinal symptoms, including ongoing haematemesis and vomiting, prompted further evaluation.

Oesophagogastroduodenoscopy revealed a large oesophageal haematoma at 20 cm (from incisors) extending to the gastroesophageal junction, approximately 10 cm in length. Contrast-enhanced CT confirmed a non-perforated lesion without mediastinal air. Clopidogrel was discontinued, and the patient was managed conservatively with intravenous proton pump inhibitors (PPIs) for 72 hours. Follow-up endoscopy at two months demonstrated complete resolution of the haematoma. This case illustrates an uncommon but important complication of antiplatelet therapy and reinforces the need for diagnostic vigilance in patients presenting with atypical gastrointestinal symptoms while on DAPT. Individualised management and multidisciplinary input are key to achieving optimal outcomes.

## Introduction

Dual antiplatelet therapy (DAPT), typically consisting of aspirin and a P2Y12 inhibitor, such as clopidogrel, is a cornerstone of secondary prevention in patients with acute coronary syndrome (ACS) and following percutaneous coronary intervention (PCI) [1]. While the benefits of DAPT in reducing thrombotic events are well-established, bleeding complications remain a major concern, particularly from the gastrointestinal tract [2]. Peptic ulcer disease and gastritis are the most common causes of upper gastrointestinal bleeding (UGIB) in this context. However, rarer causes, such as spontaneous oesophageal haematoma (SEH), may also occur and are often under-recognised [3].

Spontaneous oesophageal haematoma is a rare condition, with fewer than 100 cases described in the literature. It typically presents symptoms that may mimic more common and life-threatening conditions such as myocardial infarction, aortic dissection, or Boerhaave syndrome [4,5]. In patients receiving DAPT, oesophageal mucosa may become more vulnerable to bleeding due to impaired haemostasis and pre-existing mucosal irritation such as from reflux disease. 

Due to its rarity and overlapping symptomatology with cardiac and gastrointestinal emergencies, SEH poses a diagnostic challenge. Prompt recognition is essential to avoid unnecessary invasive interventions and to guide appropriate conservative management. We present a case of symptomatic UGIB secondary to a large oesophageal haematoma in a patient receiving DAPT, with complete resolution following the cessation of clopidogrel. This case highlights the importance of considering uncommon bleeding sites in patients on antiplatelet therapy and underscores the need for an individualised, multidisciplinary approach to managing complex presentations.

## Case presentation

We report the case of a 65-year-old woman who presented to the Emergency Department with central chest pain, radiating to the back, which started 6 hours ago. The pain was associated with nausea, vomiting, and shortness of breath. She also reported a single episode of haematemesis and described the chest pain as similar to her previous episodes of pancreatitis.

Her past medical history included PCI with drug-eluting stent insertion six months earlier for moderate right coronary artery (RCA) disease, angina, hypertension, pancreatitis, obstructive sleep apnoea (on continuous positive airway pressure (CPAP)), hyperlipidaemia, and pre-diabetes. She was otherwise fit, independent, and functionally well.

Her regular medications included aspirin 75 milligrams once a day, clopidogrel 75 milligrams once a day (post-PCI), bisoprolol 2.5 milligrams once a day, ramipril 5 milligrams once a day, isosorbide mononitrate 30 milligrams once a day (sustained release tablets), lansoprazole 15 milligrams once a day, and atorvastatin 80 milligrams once a day.

On examination, she appeared unwell and was unable to lie flat. Her abdomen was soft and non-tender. Cardiovascular and respiratory examination was unremarkable. Pulses were equal in both arms. Her heart rate was 89 per minute, blood pressure was 116/66 mmHg, and oxygen saturation was 99%. She was afebrile.

**Figure 1:**
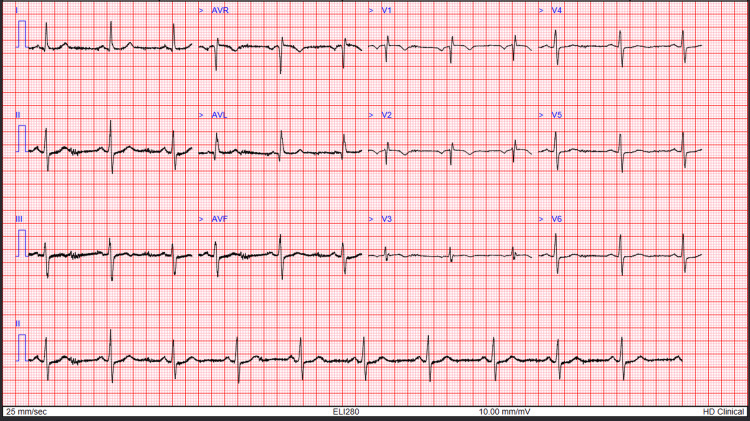
Electrocardiography (ECG) from the current presentation

**Figure 2:**
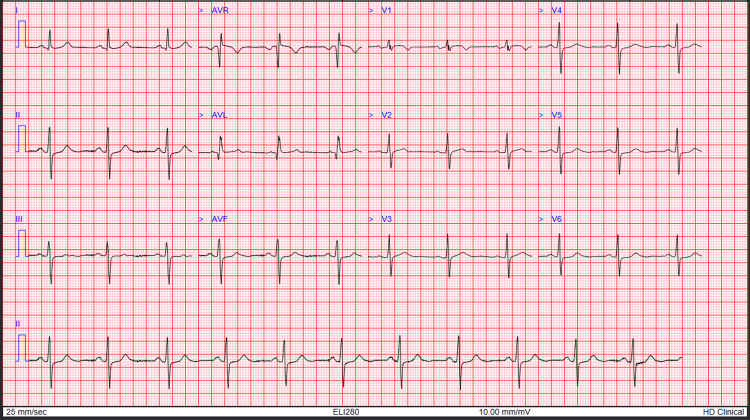
Electrocardiography (ECG) done six months prior

Electrocardiography (ECG) showed T-wave inversions in leads V1-V3 (Figure 1). Changes in lead V2 and V3 were new compared to a previous ECG done six months prior (Figure 2). Serial troponin levels were 25 and 24 ng/L (Normal <14 ng/L). Slightly elevated platelet count was deemed as reactive response, and low potassium was due to vomiting. Other laboratory investigations were unremarkable as shown in Table 1. A transthoracic echocardiogram performed on admission didn't show any regional wall motion abnormalities, and the left ventricular ejection fraction was 55%. 

**Table 1:** Initial blood investigations INR: international normalised ratio; PT: prothrombin time; APTT: activated partial thromboplastin time *Haemoglobin 2 months prior to this admission was 135 g/L.

Parameters	Results	Range	Unit
Haemoglobin*	122	(115 - 165)	g/L
White cell count	10.4	(4.0 - 11.0)	10*9/L
Platelet count	509	(150 - 400)	10*9/L
Neutrophil count	6	(1.7 - 7.5)	10*9/L
Sodium	138	(133 - 146)	mmol/L
Potassium	3.4	(3.5 - 5.3)	mmol/L
Urea	7.3	(2.5 - 7.8)	mmol/L
Creatinine	75	(45 - 85)	umol/L
INR	0.9	(0.8 - 1.2)	INR
PT	11.2	(10.3 - 13.3)	seconds
APTT	28.7	(25.7 - 35.3)	seconds
1st hs Troponin T	25	(< 14)	ng/L
2nd hs Troponin T	24	(< 14)	ng/L
Amylase	68	(28 - 100)	U/L
C-reactive protein	1	(< 5)	mg/L

**Figure 3:**
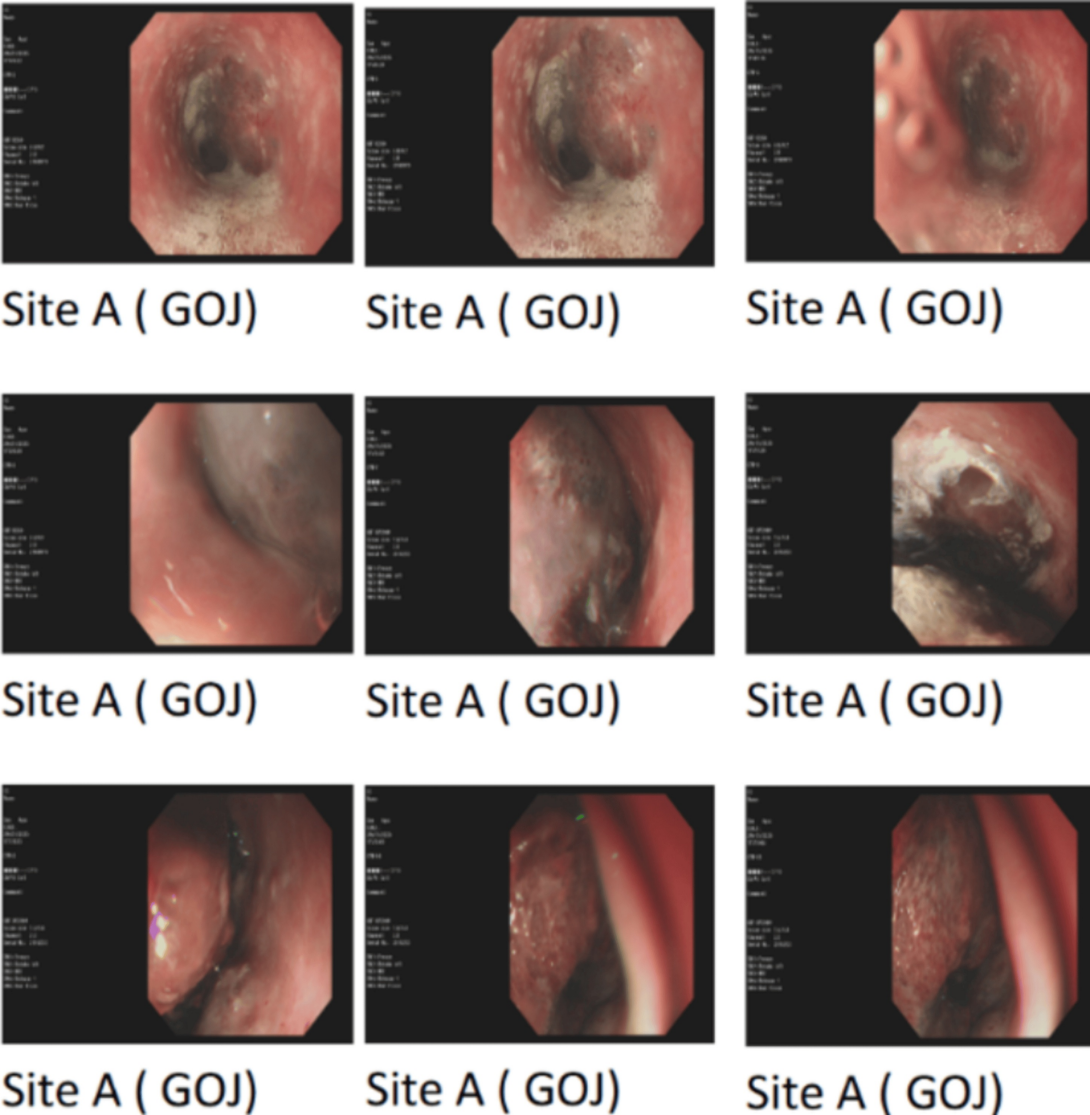
Taken at oesophagogastroduodenoscopy (OGD), suggesting an unusual finding of oesophageal lumen obliteration and the possibility of the haematoma extending to the gastroesophageal junction (GOJ)

She was initially managed as a case of suspected acute coronary syndrome (ACS) and received a loading dose of DAPT, morphine, and paracetamol. Shortly thereafter, she experienced another episode of coffee-ground vomiting, and the chest pain persisted despite the administration of morphine and paracetamol. Following consultation with a gastroenterologist, an urgent oesophagogastroduodenoscopy (OGD) was done in view of the coffee-ground vomiting and drop in haemoglobin compared to the last admission. OGD revealed a large oesophageal haematoma at 20 cm (from the incisors), extending all the way to the gastroesophageal junction, approximately 10 cm in length, with no evidence of active bleeding. The oesophageal lumen appeared obliterated as shown in Figure 3.

**Figure 4:**
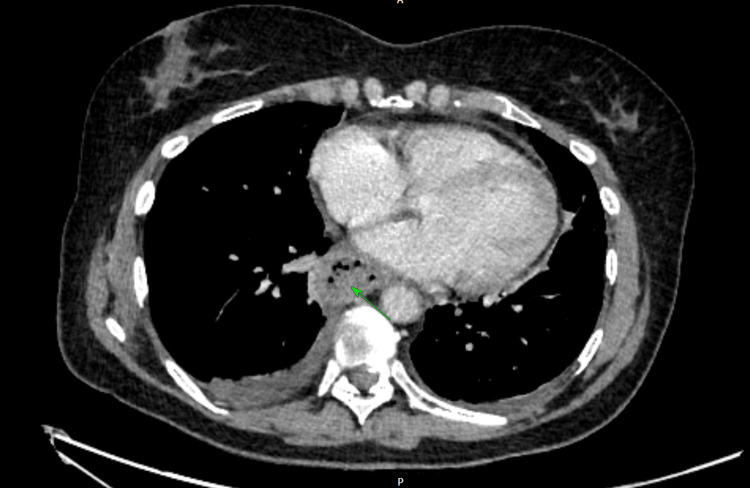
Computed tomography (CT) showing an oesophageal haematoma as pointed at by the green arrow (axial view)

**Figure 5:**
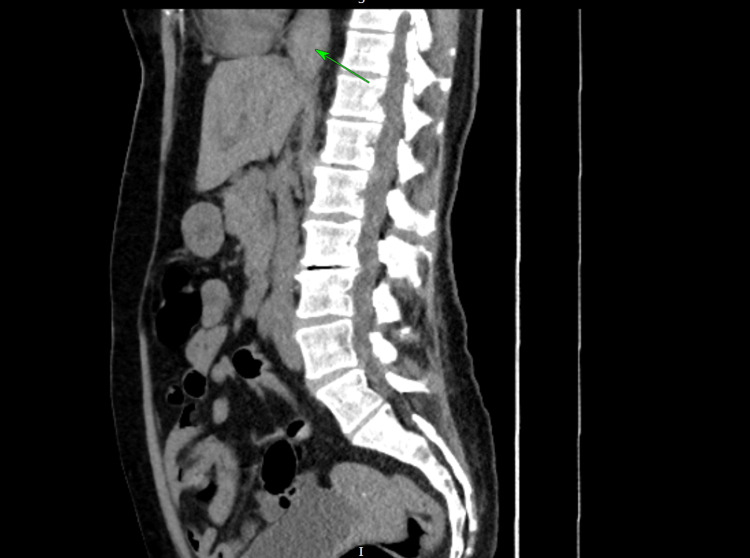
Computed tomography (CT) showing an oesophageal haematoma as pointed at by the green arrow (sagittal view)

The gastroenterology team recommended further imaging, and a contrast-enhanced computed tomography (CT) scan of the chest, abdomen, and pelvis (CAP) was performed, which confirmed a medium-sized, non-perforated distal oesophageal haematoma without evidence of mediastinal air as shown in Figures 4, 5.

The patient was managed conservatively with intravenous proton pump inhibitors (PPIs). Clopidogrel was discontinued in view of her high bleeding risk, and cardiology advised the continuation of aspirin alone at discharge, given that she had completed the guideline-recommended six-month course of DAPT.

**Figure 6:**
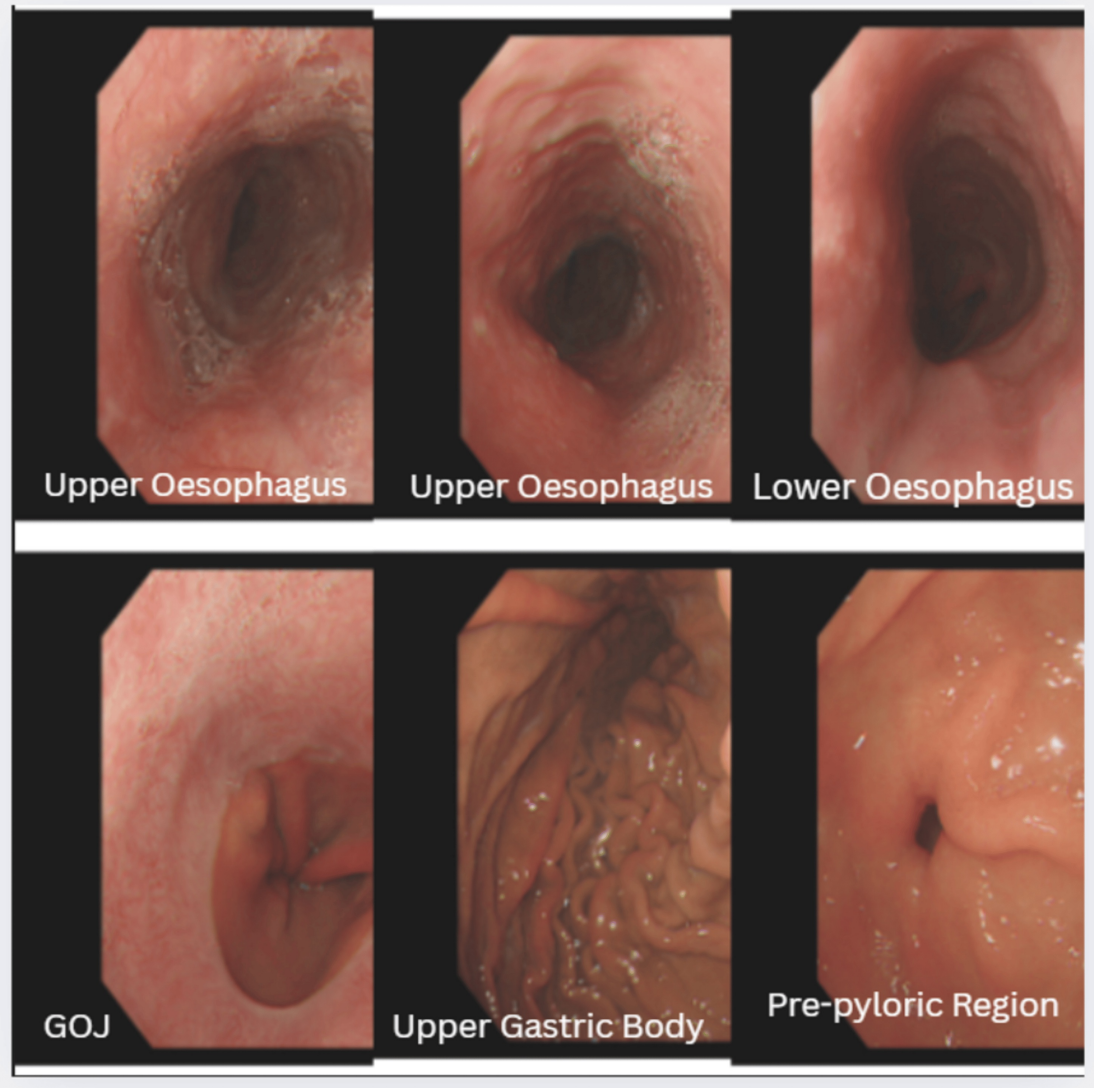
Repeat oesophagogastroduodenoscopy (OGD) demonstrating complete resolution of the haematoma

The patient’s symptoms settled after 72 hours of management, and she was safely discharged afterwards with oral PPIs. A follow-up OGD two months later demonstrated complete resolution of the haematoma and healing of the oesophageal mucosa as shown in Figure 6. 

## Discussion

SEH is a rare but important differential diagnosis in patients presenting with acute chest pain, particularly when features overlap with cardiac or gastrointestinal aetiologies. We report the case of a 65-year-old woman with a recent history of PCI on DAPT, who presented with chest pain, vomiting, with some associated difficulty in breathing, an atypical but instructive constellation of symptoms.

An initial working diagnosis of ACS was reasonable, given the patient's cardiac history, risk factors, and ECG findings of new T-wave inversions in leads V2-V3. However, cardiac biomarkers remained low and stable, and transthoracic echocardiography was unremarkable. These findings, alongside persistent symptoms and evidence of UGIB (coffee-ground emesis and haematemesis), prompted reconsideration of the initial diagnosis. 

SEH is an uncommon clinical entity, with fewer than 100 cases reported in the literature [3,6]. It is typically non-perforating and presents with retrosternal chest pain, dysphagia, odynophagia, or haematemesis, symptoms that overlap with more common conditions such as myocardial infarction, aortic dissection, or Boerhaave syndrome [4]. Recognised risk factors include vomiting, coagulopathy, antiplatelet or anticoagulant use, and oesophageal instrumentation. In our patient, likely precipitating factors included DAPT, recent episodes of forceful vomiting, and possibly mucosal fragility secondary to gastroesophageal reflux disease.

The diagnosis of SEH was confirmed via OGD, which revealed a large submucosal hematoma extending to the gastroesophageal junction. Contrast-enhanced computed tomography (CECT) provided additional details, confirming the absence of perforation or mediastinal air, and notably demonstrated indentation of the left atrium, an unusual mass effect that may have contributed to the patient’s dyspnoea, likely due to elevation in pulmonary pressures. However, no formal testing was done to confirm that. 

Management of SEH is typically conservative and includes bowel rest, intravenous PPIs, and withdrawal of contributing medications, such as anticoagulants or antiplatelet agents, when feasible [5,7]. In this case, clopidogrel was discontinued, as the patient had completed the guideline-recommended minimum six-month course of DAPT following PCI [8]. Aspirin was continued, balancing the risks of stent thrombosis and ongoing gastrointestinal bleeding. 

The hematoma resolved completely within two months, as confirmed by follow-up endoscopy, reinforcing that conservative management is effective in most cases. This case underscores the importance of maintaining a broad differential diagnosis when evaluating chest pain, especially in patients with complex medical histories and those on antithrombotic therapy. It also highlights the value of early multidisciplinary involvement, particularly from gastroenterology and cardiology, in optimising clinical outcomes.

## Conclusions

This case highlights spontaneous oesophageal haematoma (SEH) as a rare but important cause of upper gastrointestinal (GI) bleeding in patients receiving dual antiplatelet therapy (DAPT). While often misdiagnosed as more common conditions, such as myocardial infarction or peptic ulcer disease, this case underscores the importance of early endoscopic evaluation in patients on antithrombotic therapy presenting with atypical chest pain or haematemesis. Earlier consideration of endoscopy in similar clinical settings may facilitate prompt diagnosis and avoid unnecessary delays in management. Although conservative treatment remains the mainstay for SEH, this case suggests a need to re-evaluate current protocols for managing GI bleeding in DAPT patients, particularly in the acute setting, by incorporating rare complications into diagnostic pathways. Decisions regarding the continuation or adjustment of antiplatelet therapy should account not only for bleeding and thrombotic risk profiles but also for factors such as time since PCI and the type of stent used.

This case also highlights the need for close clinical follow-up and individualised risk assessment in patients who experience SEH while on DAPT. Furthermore, it supports the value of targeted education and increased awareness among emergency and cardiology teams to improve recognition and timely management of rare but serious complications like SEH.
